# Absorption of Fangs of the Temporary Teeth

**Published:** 1863-11

**Authors:** Wm. H. Atkinson


### ABSORPTION OF FANGS OF THE TEMPORARY TEETH.

BY WM. H. ATKINSON, M. D.

(Read before the N. Y. Society of Dental Surgeons.)

THE analytical mind is so much occupied with details that it fails to perceive large generalizations; while the synthetic takes so little cognizance of the minutiæ of close analysis, as to fail to practicalize its best discoveries. So we must combine broad generalization and smallness of detail, to approximate wholeness of result, and arrive at a doctrine capable of being intelligently taught to minds of small range.

The Dental profession thus far has been crippled with the former, ten times more than with the latter cause of hindrance to a rapid progress. Doubtless long and patient observation and record of the fact in minutest detail will, upon recapitulation consecutively, give the exact measure of correct synthesis of the laws of integration and disintegration.

But few have the requisite patience and perseverance, while fewer still, live long enough to have the whole range of phenomena of even a single department pass before their vision. Hence we must boldly strike off into generalizations, at the risk of going, sometimes, too far for the fractional fact-mind at once to follow us, if we would make appreciable and profitable advancement, and thus be enabled to lay aside fossilliferous weights that crush us back and counsel us to remain in the present degree of growth, lest we should see the truth, and be upheaved out of the valley of darkness, ignorance, doubt and "guessing we are right," into the blessed light of advanced demonstration and clear ability to arrange all facts so accurately as to prove the philosophy of regular growth out of primary pupilage, into the more responsible and useful status of master of both fact and principle, which are always at one with each other, to all who are enough developed to "see it."

The "carneous body" has been said to be the "cause" of the absorption of the deciduous teeth; it is rather the "agent" than the cause.

The cause producing the change of pulp into the "carneous body" under whose auspices this beautiful process is effected, is an intangible life action not easily defined.

When deficient activity is present in the breaking down and removing the obstructing fangs, there will be either arrestation of the "eruptive rise" of the forming permanent tooth, or a misdirection given to it because of this abnormity. But where there is undue acceleration of this action from any cause, there will be a receding of the crown of the deciduous tooth from the line of contact with the masticating surface of the occluding tooth in the opposing jaw, deteriorating the efficiency of the absorbing crown and its opposite in the act of mastication.

Just what arrestation of the "eruptive rise" means, will be worthy of short notice. It is a standing still of the cutting extremity of the forming tooth, without arrestation of the formative process, (i. e.) the calcification of the pulp continues to proceed, encroaching upon the sacular space, in the ratio of calciferous hardening, sometimes to the extent of completing to the apex of the fang or fangs, just beneath the external dense stratum of the jawbone, when occurring in the inferior maxillæ and encroaching upon the surrounding cancellated structures of the superior maxillæ, and even perforating, as I have often seen, the external alveolar plates in the superior maxillæ, especially witnessed in canines and first permanent molars, having no external protection but the attenuated mucous membrane, with sometimes a very little cellular membraneous tissue, scarcely concealing the bony fangs from sight.

When the saccular space is not sufficient for the full evolution of the fangs in normal line, a curvature, more or less abrupt, will occur, allowing the completion to the foramen. In such cases, as the axis of the tooth is not deflected, a well developed tooth may be erupted in normal shape, size, and position, late in life.

In fact, the tendency of all teeth is to protrude, sockets and all. when not restrained by opposing teeth in vigorous use. This projective movement is most apparent, or apparent to a greater degree in inferior, loosely organized systems; while it is absent, or scarcely discernible, in dense, compact, coarse and well knit osseous and muscular systems of many hard working people of mixed races or nationalities, whose teeth do not articulate properly, but stand cusp opposed to cusp, and space to space, preventing the teeth from coming in contact at all, until admitted by wearing down or loss of the molars.

It requires close, as well as far extended observations, to enable one to pronounce upon the abnormity or regularity of function in a given case; complete health will doubtless keep exact balance of absorption of temporary and development of permanent teeth; but there are so many modifying circumstances of type, conception, nutrition and growth, that we may find different standards of health in the same mouth even! For instance, an individual well developed, active, vigorous and happy in all respects, will shed and erupt all his teeth at the regular normal period, save the canines of the superior maxillæ, the deciduous remaining firmly set, and efficient though short and unsightly teeth. In process of time, absorption of the fangs and loosening supervene; and an increased thickening of the alveolar border presents well formed teeth of replacement, which, though late, surely come to full development, and occupy their proper places, without inconvenience other than tardiness of development.

Others will retain one or more of the temporary second molars, to ultimately give place to a well developed and properly arranged second bicuspid, etc., etc.

A condition the reverse of these last has sometimes come under my observation, that is, a premature shedding and development of the superior central incisors, without the laterals or other teeth in the mouth partaking of the precocity in any degree.

Just how much, premature absorption or extraction, has to do in hastening the eruption of the teeth of replacement, I am not able to affirm. But I do know that accidental loss of a central incisor has repeatedly seemed to hasten the development—a year or two—of its successor, in the cases referred to, while like circumstances of losses in other instances have seemed to have no effect whatever on the teeth of replacement, these coming simultaneously with their mates which had not been deprived of their predecessors.

Here again I am admonished to warn you, not to put much confidence in isolated and anomalous cases as indices of nature's mode of procedure. I wonld rather suggestively teach one dozen pupils who would thereby apprehend the principle, than hear the loud and ready response of an hundred to whom I had but "told" it to have it forgotten as as soon as out of sight.

